# Design and Manufacture of Self-Healing Asphalt Concrete with Capsules

**DOI:** 10.3390/ma19173705

**Published:** 2026-08-31

**Authors:** Sergei Inozemtcev

**Affiliations:** Building Materials Science Department, National Research Moscow State University of Civil Engineering, 129337 Moscow, Russia; inozemtsevss@mail.ru

**Keywords:** self-healing, asphalt concrete, capsules, AR-polymer, pores, pavement

## Abstract

This study focuses on the design of self-healing asphalt concrete with capsules, taking into account their size and the particle size distribution of the mineral components. A practical application of this approach is demonstrated for the production of A16U_L_ asphalt concrete with calcium alginate capsules containing a thiol-containing urethane AR polymer (ARP) for a pilot road section. The results show that self-healing efficiency depends strongly on the granulometric composition and void structure of the asphalt mixture. For the selected A16U_L_ mixture, the void network was sufficient to accommodate 1.1 mm capsules while maintaining a high capsule survival rate of 96% after compaction. Tomographic analysis confirmed a uniform distribution of capsules in the pavement structure, with less than 5% of capsules located too closely to be considered well dispersed. The use of ARP-filled capsules increased the recovery of compressive strength after healing to 38.8% of the residual strength, corresponding to 99.9% recovery of the initial strength, whereas the control mixture reached only 78.6%. In addition, the self-healing rate increased by a factor of 2.5 compared with the unmodified composition. The proposed approach demonstrates that self-healing asphalt concrete can be produced without changing the conventional production chain, provided that mixture design and capsule size are properly matched. The obtained results allow designing various types of asphalt concrete with capsules to extend the service life of pavements without repair.

## 1. Introduction

The formation of an asphalt concrete structure capable of resisting the effects of transport and weather and climatic factors is the main condition for the long-term operation of the pavement. The operational stability of the asphalt concrete structure is determined by the quality and content of materials used for its production [1,2,3,4,5,6,7,8,9,10]. The development of asphalt concrete technology contributes to the complexity of the composition of mixtures through the use of new functional modifiers, with the help of which both technological properties are regulated and materials with a unique combination of properties are synthesized.

The use of modifiers makes it possible to increase the resistance of asphalt concretes to the effects of elevated temperatures, which has a positive effect on the ability to resist dynamic loads and gauge formation in the summer period of operation [5,6,7]. Reducing the sensitivity of asphalt concrete to low temperatures makes it possible to reduce its fragility, which ensures the ability to resist cracking in winter [8,9,10].

**Figure 1:**
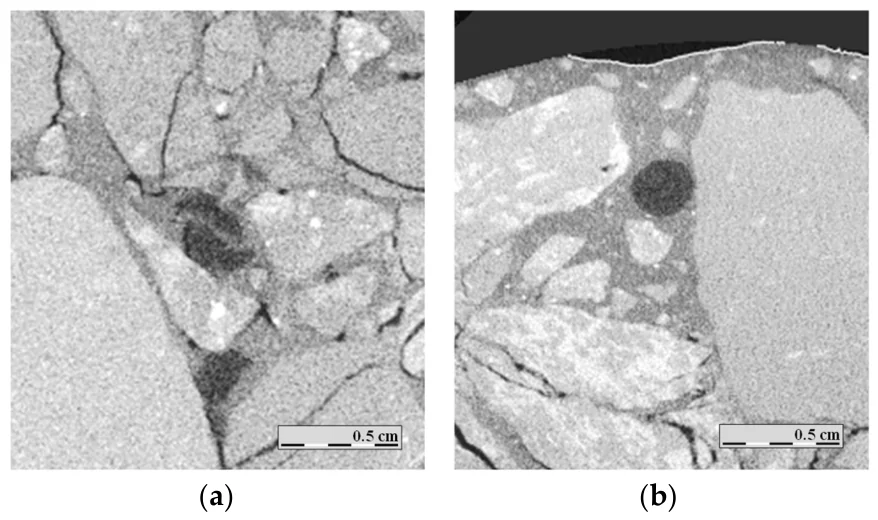
Tomographic images of the structure of asphalt concrete with premature destruction of capsules (**a**) and preservation of capsules (**b**).

The implementation of self-healing in asphalt concretes is achieved due to the resource potential formed by a special modifier in the form of capsules with a healing agent [11,12]. Capsules in asphalt concrete are an element of the structure that naturally affects the structure formation (physical and chemical properties of the surface) and the parameters of the material structure (change in the geometric characteristics of the structure: the content of components, the thickness of the bitumen layer, etc.). The purpose of the capsule shell is to preserve the healing agent from participating in structure formation processes until defects form in asphalt concrete. Therefore, exposure to other structural elements may result in a negative effect on the capsules, such as premature destruction. This can occur at the stage of production of asphalt concrete mixture and its compaction in the pavement (Figure 1).

The content of the organic and mineral part in the mixture can be different and their effect on the capsules can also be different [12,13,14,15,16,17,18,19] according to a wide range of asphalt concrete mixtures. It was previously proved [20] that in SMA-15 the preservation of capsules after compaction of the asphalt-concrete mixture reaches 97% of calcium-alginate capsules.

The preservation of capsules may be less for other types of mixtures, depending on the structure formed during compaction. The preservation of capsules in the structure of asphalt concrete depends on the function of each component of the mixture. So, in the mineral skeleton, the grains of crushed stone and coarse sand participate in the formation of a rigid spatial frame, in which the grains, when in contact with each other, form zones of the greatest mechanical stress during compaction [21,22]. When entering such zones, the capsules experience heavy loads and can be destroyed.

Fine fractions of sand and mineral filler together with bitumen form asphalt binder, which fills space between grains of rigid spatial frame. Significantly less pressure acts on the capsules through the asphalt binder as a result of its distribution. This enhances the integrity of the capsules in such an environment during densification.

Thus, the purpose of this work is to establish the boundary conditions for the use of a modifier in the form of capsules with a healing agent in asphalt concrete mixtures with a different particle size distribution and amount of bitumen binder, as well as industrial testing of their use.

## 2. Materials and Methods

Grain compositions of the mineral skeleton of crushed stone-mastic asphalt concrete mixtures in accordance with the standard GOST 31015-2002, GOST R 58406.1-2020 and GOST R 58401.2-2019 [23,24,25], as well as hot asphalt concrete mixtures in accordance with the national standard GOST 9128-2013, GOST R 58406.2-2020 and GOST R 58401.1-2019 [26,27,28] were studied. Features of the structure of mixtures and asphalt concretes, taking into account the Voids in Mineral Aggregate (VMA), voids filled with asphalt (VFA), bulk specific gravity of the combined aggregate blend and air voids were studied.

**Figure 2:**
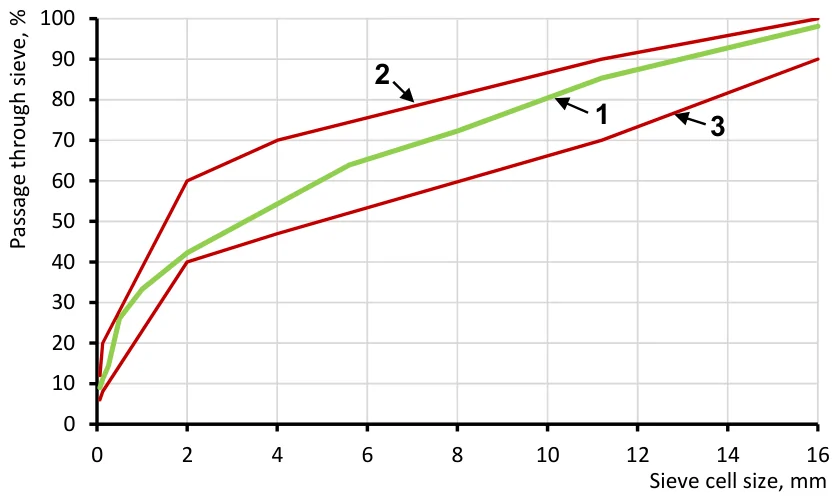
Grain composition of asphalt concrete mix A16U_L_ (1), upper (2) and lower boundary (3).

Asphalt concrete mixture with a nominally maximum size of 16.0 mm mineral aggregate (A16) used for the top layer (U) of the coating with light traffic conditions (L) was A16U_L_ considered for industrial production. A16U_L_ grain composition is designed in accordance with state standard GOST R 58406.2-2020 (Figure 2).

Crushed stone of the granite fraction of grains from 4 to 16 mm with the grade of M1200 crushability (LLC TD Uraldolomite: Ekaterinburg, Russia), corresponding to the requirements of state standard GOST 32703-2014 [29] was used as a coarse aggregate. Crushed granite sand grain size from 0 to 4 mm with the grade of M1200 crushability (LLC TD Uraldolomite: Ekaterinburg, Russia), and enriched sand (Stroysyrye LLC: Kazan, Russia), the corresponding requirements of state standard GOST 32730-2014 [30] were used as fine aggregate. Activated mineral powder of MP-1 grade (Tatavtodor JSC: Kazan, Russia) meeting the requirements of state standard GOST 32761-2014 [31] was used as filler.

BND 70/100 oil road bitumen (Taif-NK JSC: Nizhnekamsk, Russia) meeting the requirements of state standard GOST 33133-2014 [32] was used as binder. The optimum bitumen content of the mixture was 4.19% over 100% by weight of the mineral components. The main properties of bitumen are presented in Table 1.

**Figure 3:**
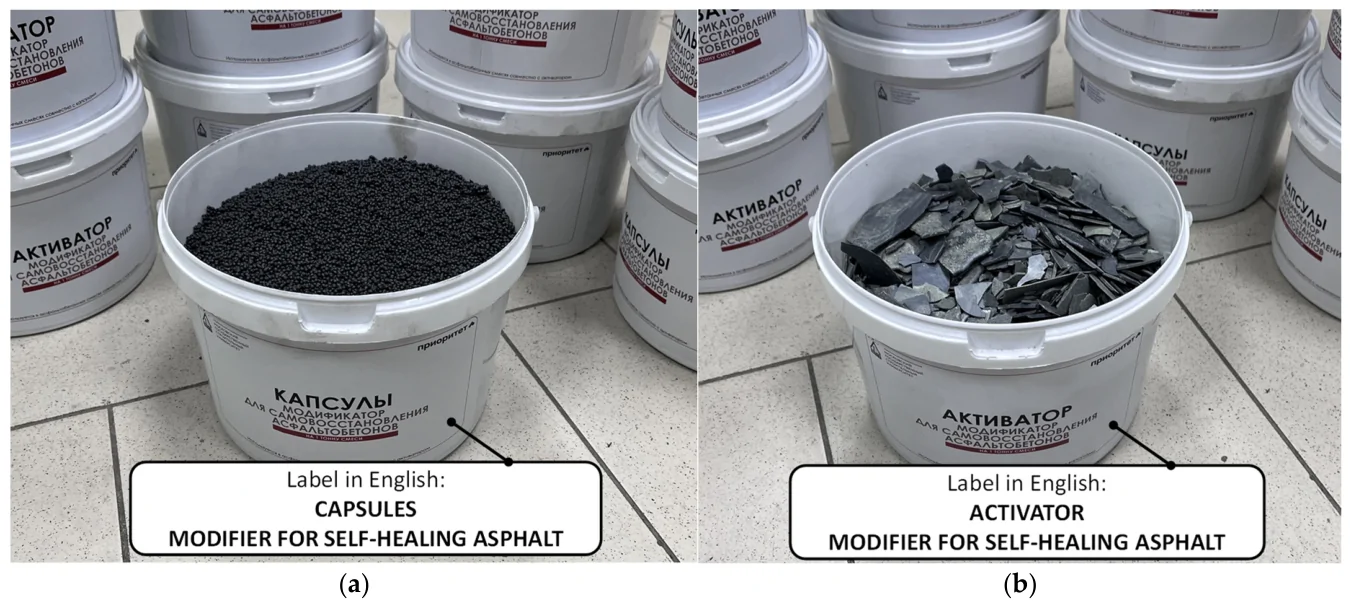
Appearance of capsules with capsules (ARP) (**a**) and activator (**b**).

Calcium alginate capsules with a diameter of 1.0–1.1 mm, strength–18 N, heat resistance–150 °C, containing AR-polymer (ARP) [20] (LLC PoliMix Kazan: village Rozhdestveno, Republic of Tatarstan, Russia) at least 90% of the volume were used as a modifier for self-healing of asphalt concrete (Figure 3a). A mixture of industrial sulfur (Gazprom Dobycha Orenburg LLC: Orenburg, Russia) and manganese (II) oxide (LLC Khimbaza: Moscow, Russia) in a ratio of 2:1 (Activator) was used to activate the polymerization of the AR-polymer [20] (Figure 3b).

The composition of asphalt concrete is presented in Table 2.

Asphalt concrete mixture of A16U_L_ modified by capsules for self- healing was produced at the Tatavtodor JSC (Kazan, Russia) plant using the AMMANN GLOBAL asphalt mixer. The preparation was carried out according to the traditional technology at the mixing temperature of the components 141 ± 1 °C:

- mixing of mineral components;
- adding bitumen and stirring for 20 s;
- adding of activator and stirring for 20 s;
- adding of capsules and stirring for 20 s;
- shipment to the collecting hopper.

**Figure 4:**
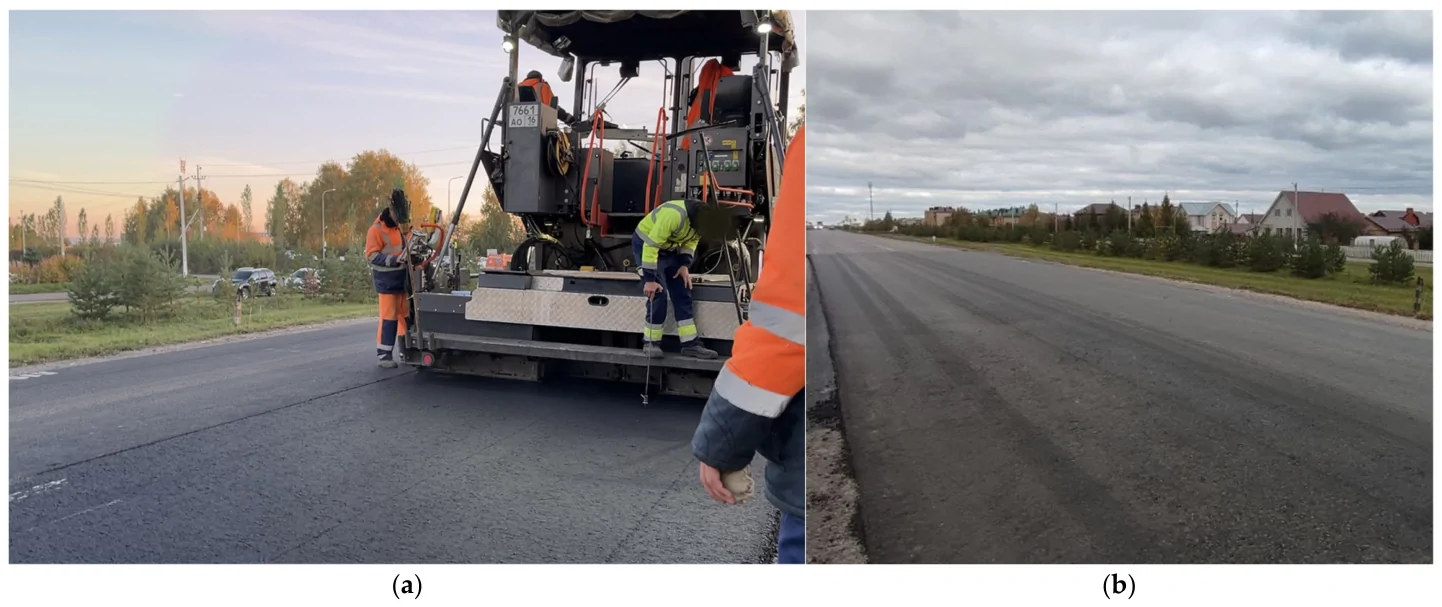
Photo of the process of laying the mixture (**a**) and the finished pavement (**b**).

The mixture was transported at a distance of 52 km from the manufacturer to the laying site in the village of Pestretsy of the Republic of Tatarstan on the section of the road “Old Shigaleevo-Pestretsy-Lenino-Kokushkino” using the Graber thermal container. The asphalt concrete mixture with self-healing capsules was laid in clear weather (air temperature +8 ± 1 °C, no precipitation) using a Volvo P7820D auto-layer and compacted using Bomag BW 151 AD-4 and Hamm HD 90 rollers at a mixture temperature of 131 ± 1 °C (Figure 4).

The properties of asphalt concrete with capsules and control composition were evaluated on core samples from the coating and samples made from the mixture taken from the mixer. The main properties of A16U_L_ asphalt concrete with capsules are presented in Table 3.

The self-healing index for the change in the ultimate indirect tensile strength at a temperature of 5 °C in accordance with state standard GOST R 58401.18-2019 [33] was determined for core samples. The split cores were stored in the laboratory at a temperature of 20 ± 2 °C for 14 days, after which they were retested.

21 sample cylinders with a diameter of 71.4 mm were prepared from each selected mixture, which were tested for compression at a temperature of 20 °C. One set of cylinder samples was retested after compression to determine residual strength, and the remaining samples were stored in the laboratory at 20 ± 2 °C for 14 days. A repeated compression test after self-healing was carried out after 1 day, 3 days, 7 days and 14 days.

The result of self-healing of asphalt concrete was estimated by calculation using the formulas:

(1) $$HI=\frac{R_{hi}}{R_{0}}$$

(2) $$SHI=\frac{R_{hi}-R_{res}}{R_{res}}$$

where *R*_0_ is initial ultimate strength, MPa; *R_res_* is residual strength after determination of strength, MPa; *R_hi_* is strength after self-healing after *i* days, MPa.

Specimens tested for compressive strength were used to determine residual strength. The compression of the samples only led to the rupture of part of the bonds in the sample and the violation of its continuity (the formation of defects). These samples were retested for compressive strength. Based on these data, residual strength was calculated. The *HI* coefficient characterizes the degree of healing of the structure of asphalt concrete after self-healing, and the *SHI* coefficient–the multiplicity of the increase in the strength of the material after healing compared to the residual strength.

The uniformity and distribution of capsules in the volume of asphalt concrete was carried out on core samples taken from the coating using a Hamamatsu L12161-07 tomograph. X-ray radiation was used with an anode voltage of 150 kV, a tube current of 180 μA, a focal spot size of 50 μm at a distance of 314.8 mm from the source to the object, and 536.4 mm from the source to the detector. 3 frames for averaging over 3600 projections were used to obtain an image.

**Figure 5:**
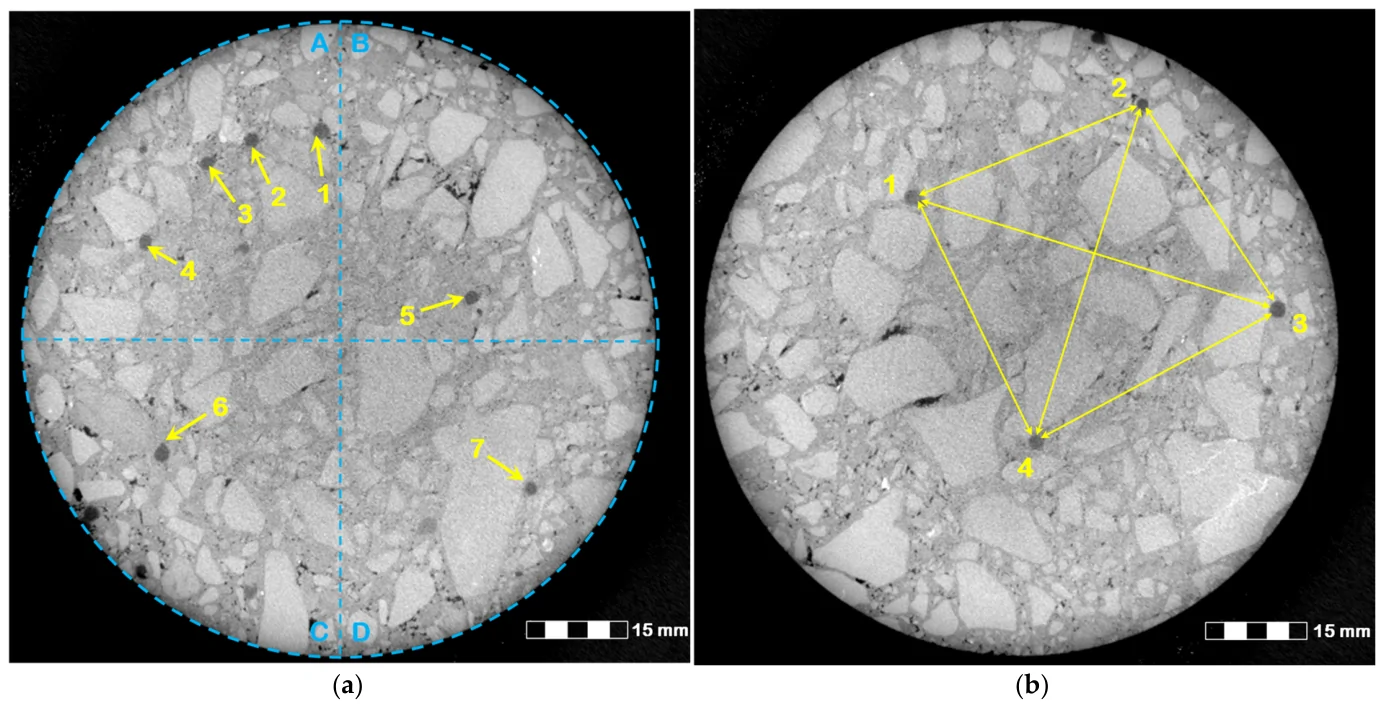
Scheme for determining the number of capsules (**a**) and the distance between capsules (**b**).

The projection every 2 mm was divided into 4 equal sectors A, B, C and D, in which whole and destroyed capsules were counted to assess the distribution of capsule in the volume of samples (Figure 5a).

The distance between capsules using the myVGL software was measured in a projection every 2 mm to assess the uniformity of capsules in the volume of samples (Figure 5b).

Measures of central trend and measures of variability, as well as Fisher’s test variability, were used to process empirical data.

## 3. Results and Discussion

Preservation of capsules in the asphalt concrete structure after compaction of the mixture is achieved by meeting two basic conditions:

– mineral framework with minimum concentration of high mechanical stress zones;
– sufficient amount of asphalt binder.

The condition for forming the mineral skeleton in which the capsules do not collapse is to form a structure where the capsules do not disturb the natural packaging of the grains and do not act as a skeleton element.

**Figure 6:**
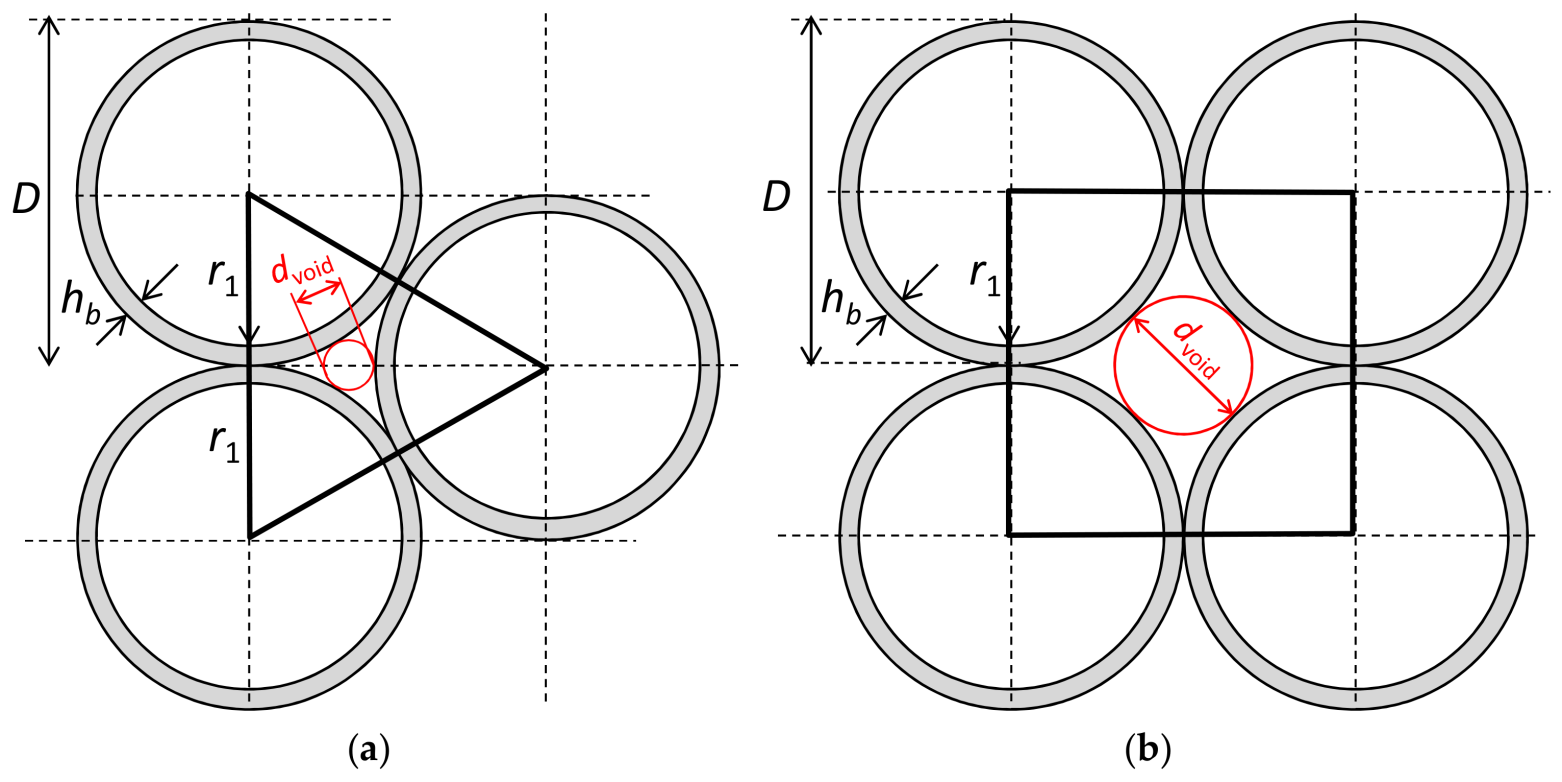
Model for calculating the dimensions for placing capsules in a grain core for face-centered cubic (**a**) and cubic grain packing (**b**).

Considering the cubic and face-centered packaging of monodisperse grains of spherical shape with diameter *D*, the size of the void between the particles, where the additional element can be placed without expanding the grains, is 0.414∙*D* and 0.155∙*D*, respectively (Figure 6).

In such a case, the value characterizing the dimensional boundary of the objects influencing the packing of the grains of the mineral framework is 0.285∙*D*. Thus, if a capsule with a diameter *d_c_* < 0.285∙*D* is added to the mixture, it is freely placed in the intergranular space and is in a volume surrounded by bitumen and small particles of the mineral part, with a distributed pressure from the compaction of the mixture. If the diameter of the capsule *d_c_* ≥ 0.285∙*D*, then the capsule becomes an element of the mineral framework, integrating into the chain of grain contacts through which compressive and shear loads are perceived with further concentration of stresses. As a result, the capsule can be destroyed.

It should be borne in mind that the mineral composition of the asphalt concrete mixture consists of a polydisperse mixture of angular grains, which are laid in an irregular order. The minimum grain size of the mineral framework will differ for different types of asphalt concrete mixtures. The size in the mineral framework depends on the distance between particles, which is calculated based on the formula [34,35,36]:

(3) $$h\approx \frac{{\langle d^{3}\rangle -\langle d^{2}\rangle }^{3/2}}{\langle d^{2}\rangle }$$

where $\langle d^{2}\rangle$ and $\langle d^{3}\rangle$ are weighted average of the particle diameter in the polyfraction mixture, with exponents of two and three, respectively; $\langle d^{n}\rangle =\sum_{i}f_{i}\cdot d_{i}^{n}$; *f_i_* is volume fraction of grains with diameter *d_i_*.

The numerator of the formula is proportional to the free volume in the particle package, and the denominator is the total surface of the particles. In an ideal system, the average radius of the voids or cavity where the capsule can be placed will be 0.5∙*h*, then for an asphalt concrete mixture this size will be much smaller. Then the radius of the voids or the permissible size of the capsules in the asphalt concrete mixture will be equal to

(4) $$2\cdot r_{voids}=k\frac{{\langle d^{3}\rangle -\langle d^{2}\rangle }^{3/2}}{\langle d^{2}\rangle }$$

for which *k* depends on several parameters:

(5) $$k=k_{poly}\cdot k_{form}\cdot k_{bit}\cdot k_{pack}$$

where *k_poly_*, *k_form_*, *k_bit_*, *k_pack_* < 1.0 are coefficients taking into account the polydispersity of the grain composition, the non-spherical shape of the grains, the presence of bitumen in the system, the heterogeneity of packaging, respectively.

The polydispersity coefficient can be calculated using the number-average diameter *d_n_* and mass-average (or volume-average) diameter *d_w_* [37,38]:

(6) $$k_{poly}=\frac{{\bar{d}}_{n}}{{\bar{d}}_{w}}$$

for which:

(7) $${\bar{d}}_{n}=\sum_{i}f_{ni}d_{i}$$

where $f_{ni}=n_{i}/\sum_{i}n_{i}$ is fraction of the *i*-th fraction (diameter *d_i_*); *n_i_* is the number of particles of the *i*-th fraction;

(8) $${\bar{d}}_{w}=\sum_{i}f_{wi}d_{i}$$

where $f_{wi}=n_{i}d_{i}^{3}/\sum_{i}n_{i}d_{i}^{3}$ is mass (volume) fraction of the *i*-th fraction (diameter *d_i_*).

In accordance with [39,40], the shape coefficient for spherical particles *k_form_* = 1, and for the rounded and angular shape of the particles, which is characteristic of the grains of asphalt concrete aggregate *k_form_* = 0.77 and *k_form_* = 0.66, respectively. The presence of low quality oblong and lamellar grains reduces *k_form_* to 0.58 and 0.43, respectively.

Obviously, the introduction of bitumen into the mineral part helps to reduce the volume of *V*_0_ voids to a certain value *V*_1_, which, taking into account the assumption of their spherical shape [16], is associated with their radius as a function of $r^{3}\propto V$ or $r\propto V^{1/3}$. Then the change in the size of the voids will be determined taking into account the voids filled with *VFA* bitumen [41]:

(9) $$k_{bit}=\frac{r_{1}}{r_{0}}=\sqrt[{3}]{\frac{V_{1}}{V_{0}}}=\sqrt[{3}]{1-VFA}$$

Voids in mineral aggregate in asphalt-concrete mixture is on average equal to 12–18%, which makes it possible to use coefficient of grains packing *k_pack_* from 0.82 to 0.88.

Thus, for the types of asphalt concrete mixtures, structural parameters can be determined to judge whether capsules can be used, such as the average number of contacts per grain and the average size of voids between grains. Some properties of standard asphalt concrete mixtures are presented in Table 4.

Using formula (5), the studied asphalt-concrete mixtures were evaluated by the parameter *d_voids_* = 2∙*r_voids_*. The calculation results are presented in Table 5.

Analysis of grain compositions of various types of asphalt concrete mixtures shows that these mixtures have the necessary structure to accommodate self-healing capsules with a diameter of 1.1 mm only when using aggregate with a maximum size of at least 10 mm. These mixtures include crushed stone-mastic asphalt concretes SMA-20 and SMA-15 (GOST 31015), SMA22, SMA16 and SMA11 (GOST R 58406.1), SMA-22 and SMA-16 (GOST R 58401.2), as well as asphalt concretes from hot mixtures A22U_L_ and A16U_L_ (GOST R 58406.2), SP-32, SP-22 and SP-16 (GOST R 58401.1). It is also important to note mixtures of type A, type B, type B, A11 and SP-11, in which, with some grain compositions near the maximum permissible border, voids can form in the frame with sufficient volume to accommodate capsules.

**Figure 7:**
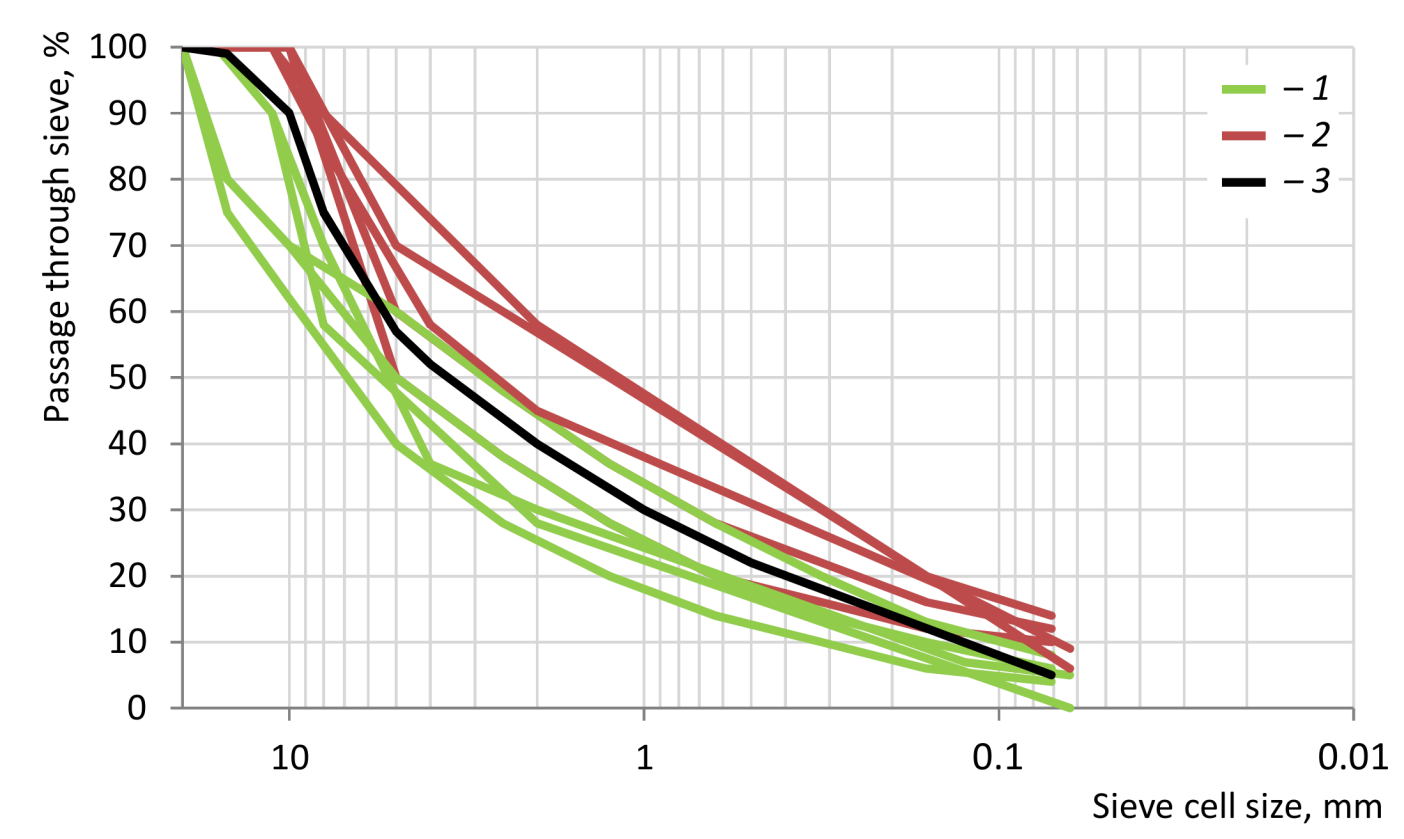
Particle size distribution of asphalt concrete mixtures with void sizes: 1—*d_v_* > 1.1 mm; 2—*d_v_* < 1.1 mm; 3—conditional boundary.

Thus, the analysis of regulatory requirements for the granulometric composition of asphalt concrete mixtures ensured the establishment of a boundary between the concentrations of various fractions of mineral components, with the help of which a mineral framework is formed with voids between the grains, in which capsules for self- healing of asphalt concrete can be placed. Grain compositions of asphalt concrete mixtures are presented in Figure 7.

Analysis of Figure 5 shows that for asphalt concrete mixtures, within acceptable limits, the mineral mixture can be selected for the use of capsules (with a diameter of 1.1 mm), as well as compositions in which they will be destroyed during compaction. The recipe boundary of grain compositions for such mixtures relative to which its suitability for the implementation of self-healing technology is assessed is proposed (Table 6).

The established boundary can be used to assess the composition of the mineral part of the asphalt concrete mixture for the use of capsules with a diameter of 1.1 mm. For capsules of other sizes, the grain size limit shall be specified.

**Figure 8:**
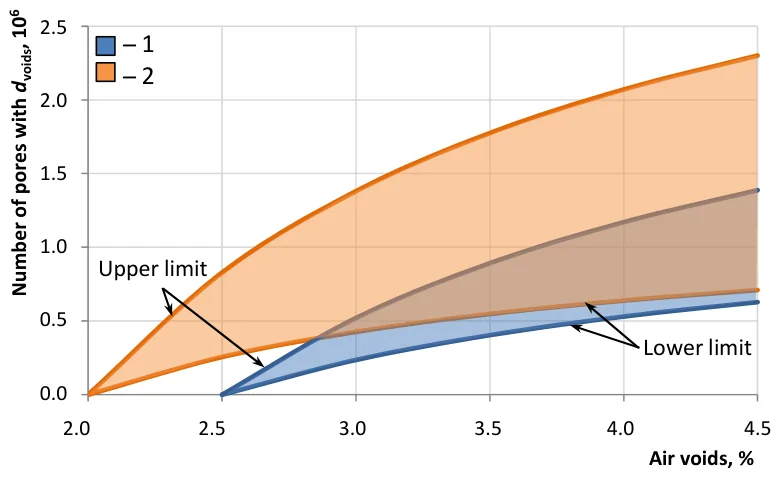
Dependence of the number of pores for 1 m^3^ of A16U on the residual porosity for: 1—severe traffic conditions (more than 1.8 million vehicles); 2—light traffic conditions (less than 0.5 million vehicles).

The optimal structure of asphalt concrete after compaction of the mixture is characterized by residual porosity, which is regulated by the relevant standard. The addition of capsules to the asphalt concrete mixture leads to the fact that they can occupy the volume of intergranular voids, reducing the residual porosity (with a constant flow of bitumen and mineral powder). It follows that the number of pores that can be occupied by capsules is limited by the minimum possible residual porosity (Figure 8).

When the residual porosity of 2.0% and 2.5% are reached, there are no pores in the A16U structure for the light and severe traffic conditions with the size of *d_voids_* that can be occupied by capsules, which will lead to their displacement and possible destruction of the aggregate grains when compacting the asphalt concrete mixture. Figure 8 shows that within the specified limits, an increase in residual porosity is a condition for an increase in the number of pores suitable to be filled with capsules with a diameter of 1.1 mm. The maximum capsule content (% by weight of bitumen) can be calculated using the formula:

(10) $$C_{cap,max}=\frac{\rho_{cap}}{\rho_{bit}}\cdot \frac{\left(P_{a}-P_{a,min}\right)}{VMA\cdot VFA}{\cdot 10}^{4}$$

where ρ*_bit_* and ρ_cap_ are density of bitumen and capsules with healing agent, respectively, kg/m^3^; VMA is mineral aggregate voids; *P_a_* is residual porosity of asphalt concrete, %; VFA is total number of voids filled with binder, expressed as a percentage of the volume of voids in the mineral aggregate, %; *P_a_*_,min_ is minimum residual porosity of asphalt concrete in accordance with state standard requirements.

**Figure 9:**
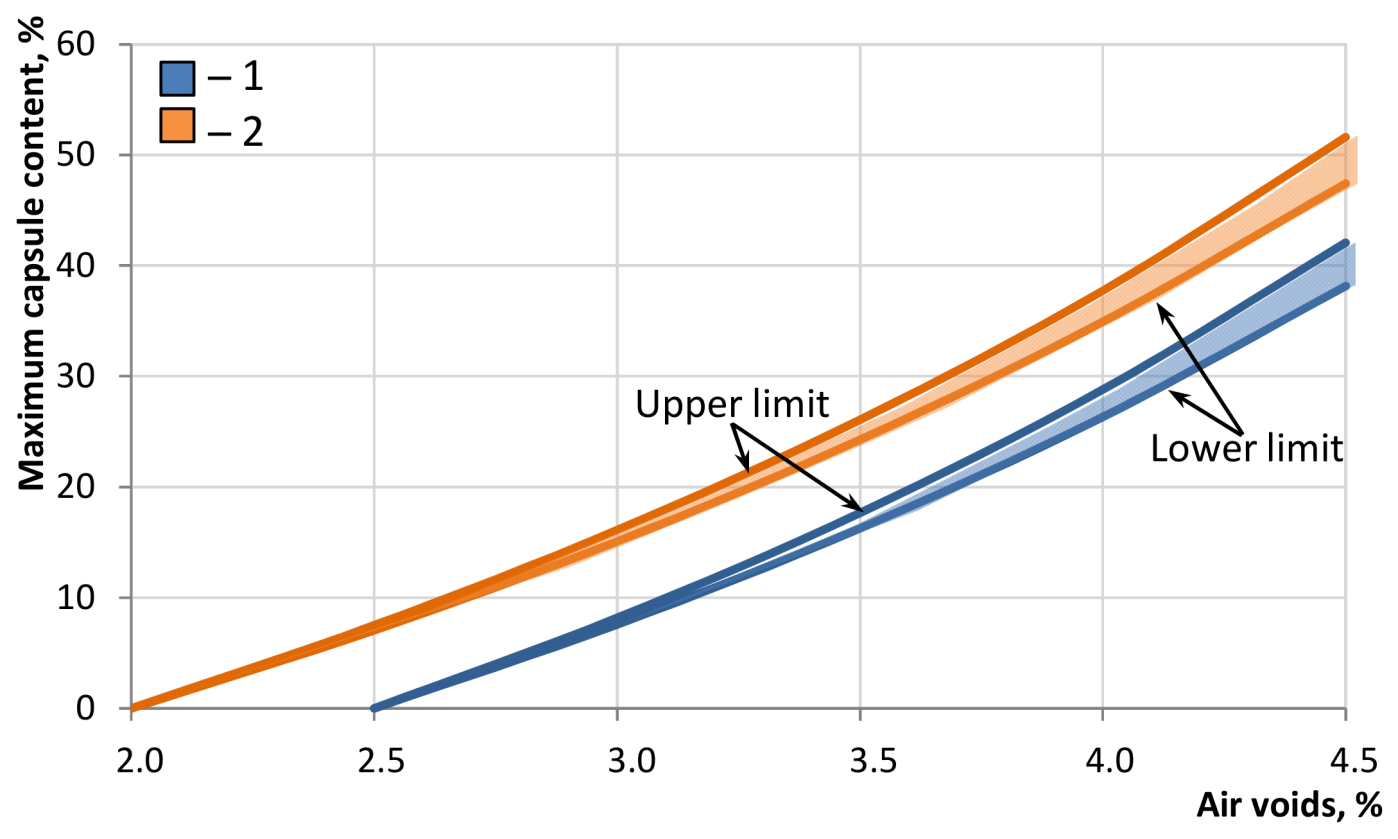
Dependence of the maximum consumption of capsules per 1 m^3^ of A16U on the residual porosity for: 1—severe traffic conditions (more than 1.8 million vehicles); 2—light driving conditions (less than 0.5 million vehicles).

This condition can be used when designing an asphalt concrete composition having self-healing [42], which is provided by the introduction of capsules containing a healing agent (Figure 9).

Figure 9 show that an increase in residual porosity of every 1% increases the potential flow rate of capsules from *d_voids_* by 15–18%. This result makes it possible to optimize the number of capsules in the mixture in terms of self-healing efficiency. Thus, the Dependence of the maximum consumption of capsules per 1 m3 allows, when designing a mixture, to determine their permissible concentrations, which are justified and depend on the structure of the formed mineral framework.

The particle size distribution for the A16U_L_ can be estimated taking into account the obtained conditions. The polydispersity coefficient calculated in accordance with [18,19,20] for the grain composition under study is *k_poly_* = 0.38. The coefficient taking into account the non-spherical shape for the studied asphalt concrete mixture is taken as *k_form_* = 0.66 for grains of the angular mineral framework [21]. Based on the number of pores filled with bitumen, the coefficient *k_bit_* = 0.65 is A16U_L_ for the selected composition, and the coefficient of heterogeneity of packing the grains of the mineral framework is *k_pack_* = 0.84.

**Figure 10:**
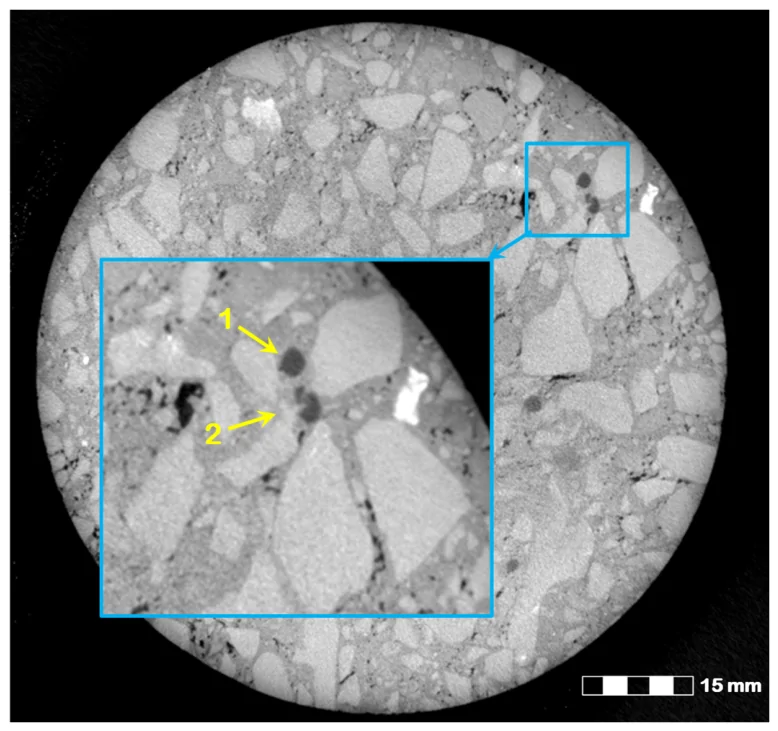
Tomographic image of asphalt concrete core with whole (1) and broken capsule (2).

In asphalt concrete A16U_L_ with a grain composition shown in Figure 2 and a bitumen content of 4.19%, voids with a diameter of 1.3 mm are formed, in which capsules can be placed, which is 20–30% more than the diameter of the capsules used. Thus, the asphalt concrete mixture is A16U_L_ suitable for self-healing technology. Cores were taken from the asphalt concrete coating with capsules to study the structure (Figure 10).

Tomographic studies showed that only 4% of the capsule containers collapsed as a result of pinching between the grains of large aggregate. This result correlates well with the results previously obtained using numerical modeling [20].

**Figure 11:**
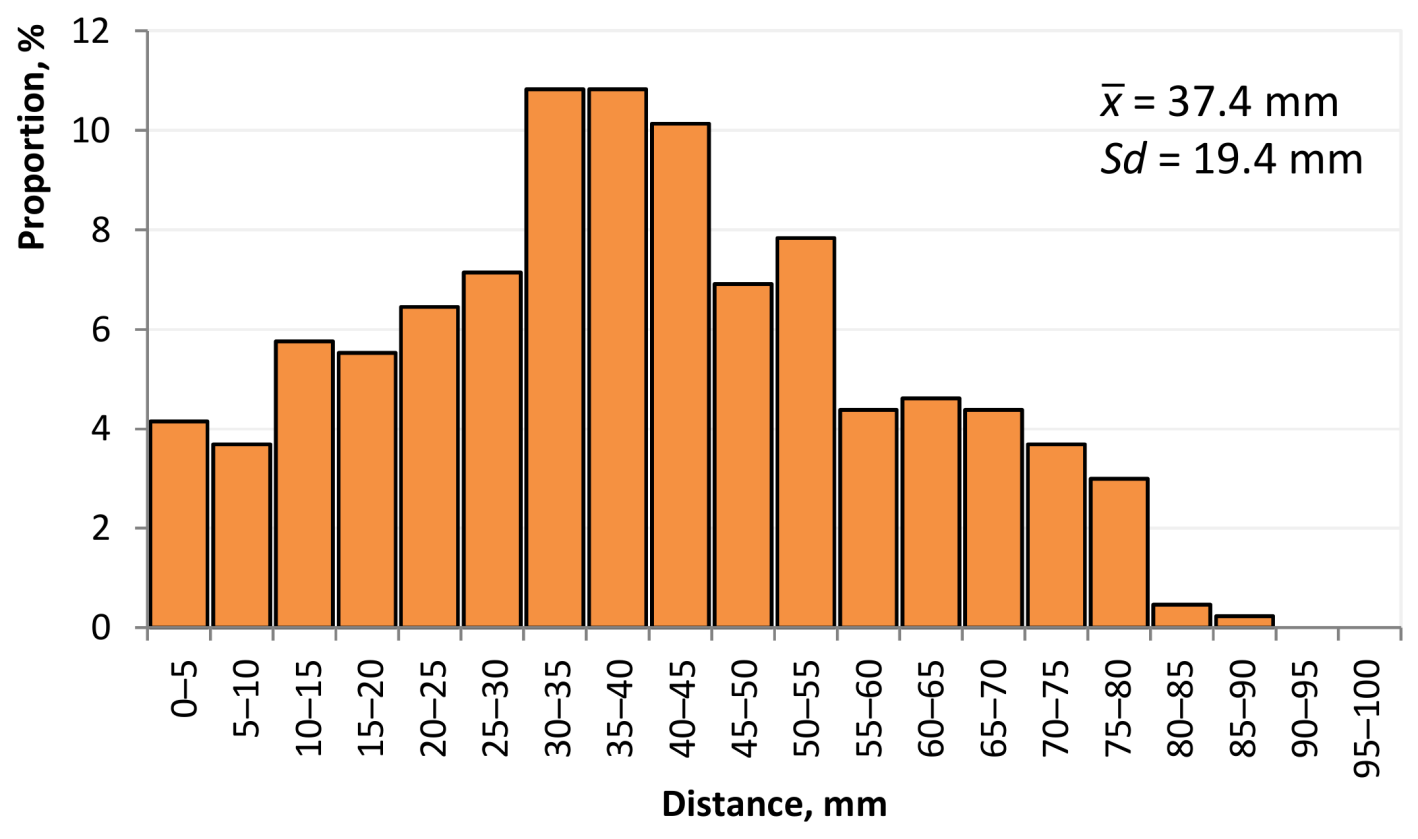
Histogram of spacing between capsules in core samples.

The variability of capsule distribution in the volume of the material is confirmed by the fact that in each quarter of the volume of the studied core samples there are from 22 to 28% of capsules, with a standard deviation of 3% and an overall variability according to Fisher’s criterion F = 0.76 (Figure 11).

The histogram of the distribution of distances between capsules in core samples (Figure 11) indicates the uniformity of the distribution of capsules in the volume of core samples, which are located on average at a distance of 3.9 cm from each other. Less than 5% of capsules can be considered not distributed, which are located at a distance of less than 0.5 cm from each other.

**Figure 12:**
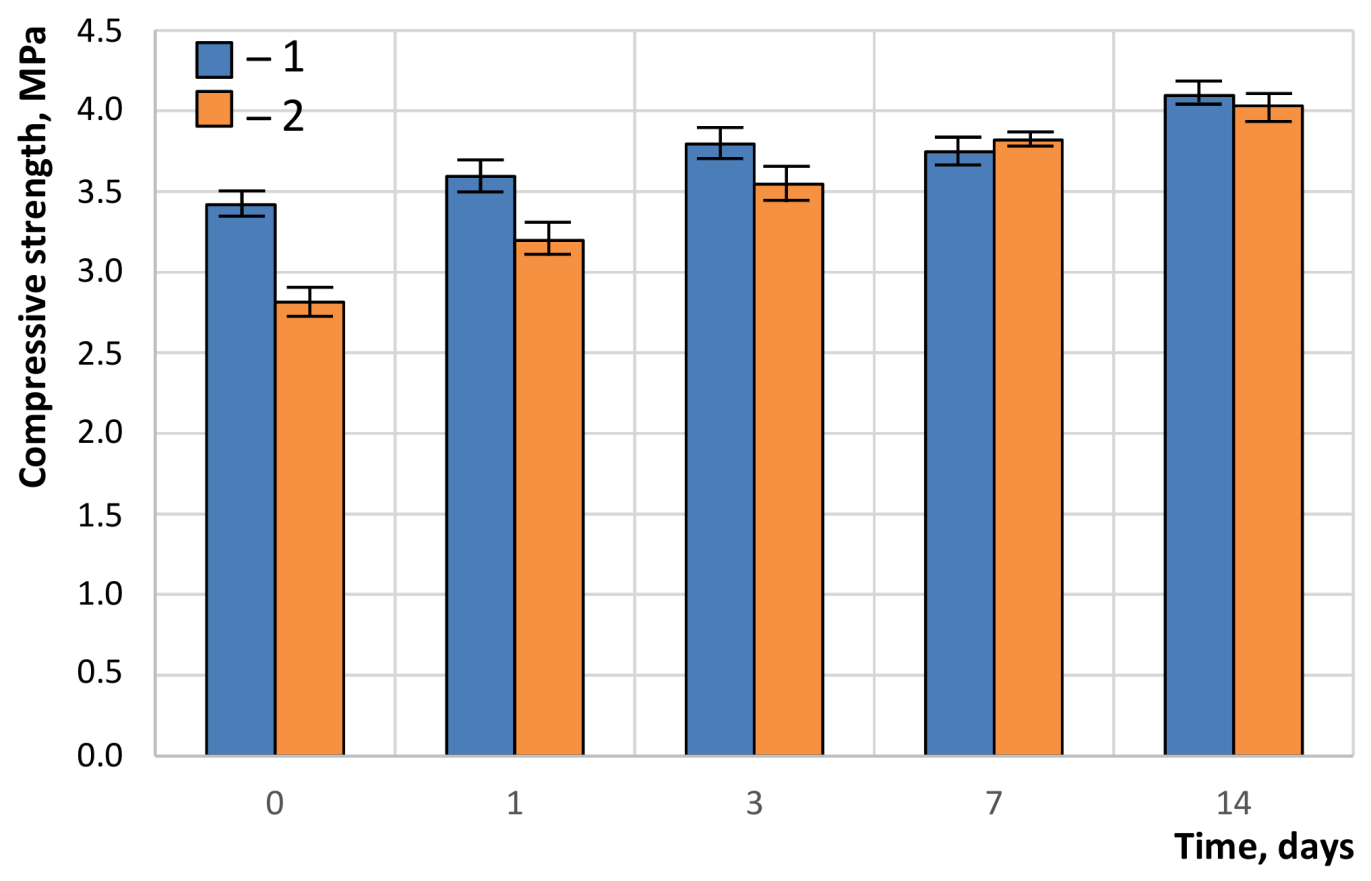
Change in compressive strength of asphalt concrete of ordinary composition (1) and with capsules (2).

Structural studies show that the distribution of capsules in the volume of asphalt concrete can ensure a uniform implementation of the self-healing mechanism. Thus, it was found that the self-healing *HI* for the breaking strength of core samples of the control composition is 20%, while the use of capsules with an AR polymer can increase this figure by 2.2 times to 44%. The results of determination of ultimate compressive strength of asphalt concrete samples are shown in Figure 12.

**Figure 13:**
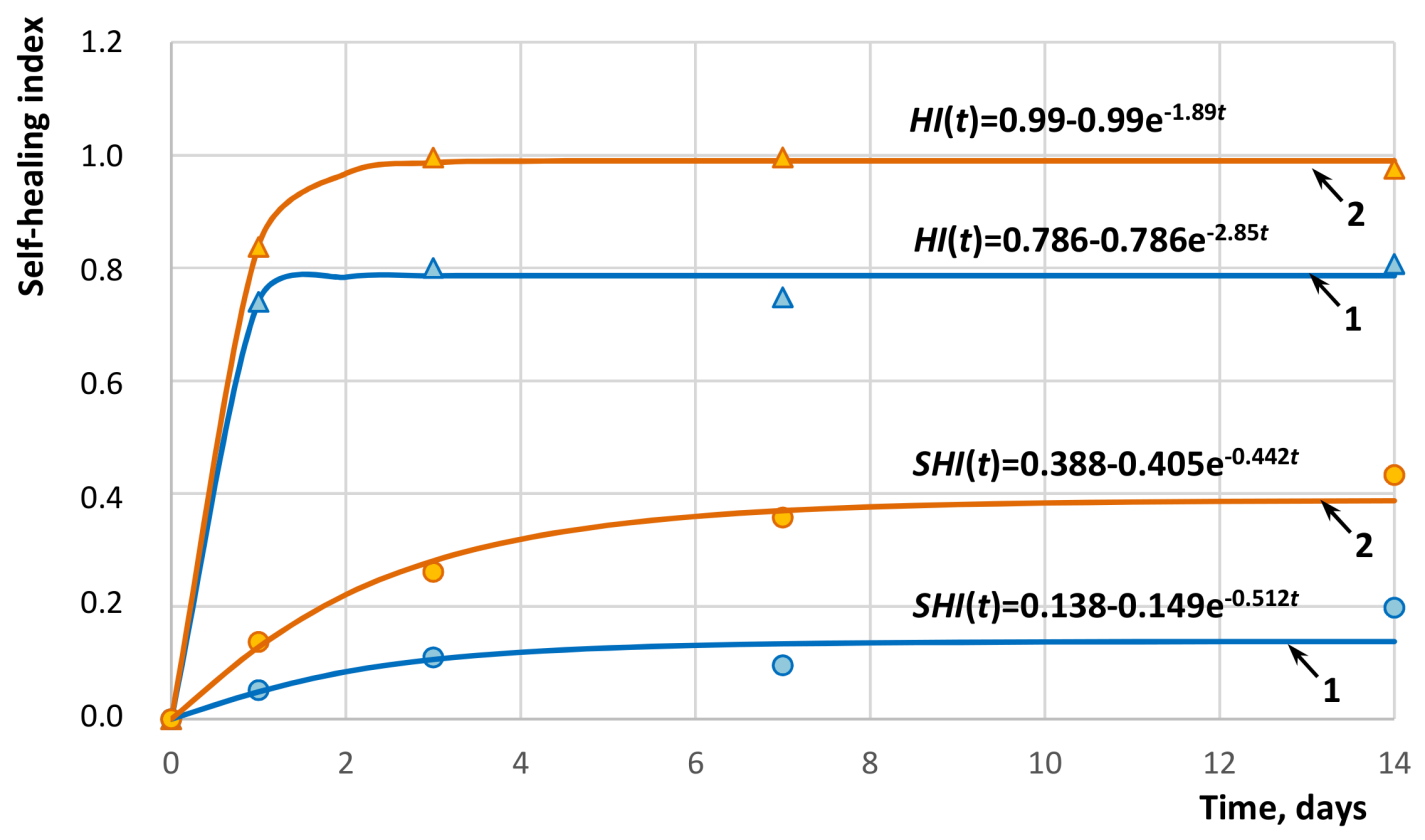
Change in the self-healing factor *HI* and *SHI* of asphalt concrete of ordinary composition (1) and with capsules (2).

Analysis of the data shows that within 14 days the structure of asphalt concrete is restored to varying degrees. Despite the lower initial strength of asphalt concrete with capsules, the self-healing processes using AR-polymer increase strength more effectively than in traditional asphalt concrete. As a result, after 14 days, the strength of the two types of asphalt concrete does not differ significantly. Variation of self-healing coefficients *HI* and *SHI* according to equation:

$${SHI}_{i}\left(t\right)={SHI}_{max}-ae^{-bt}$$

here a and b are empirical coefficients show that more self-healing is realized in asphalt concrete A16U_L_ with capsules (Figure 13).

**Figure 14:**
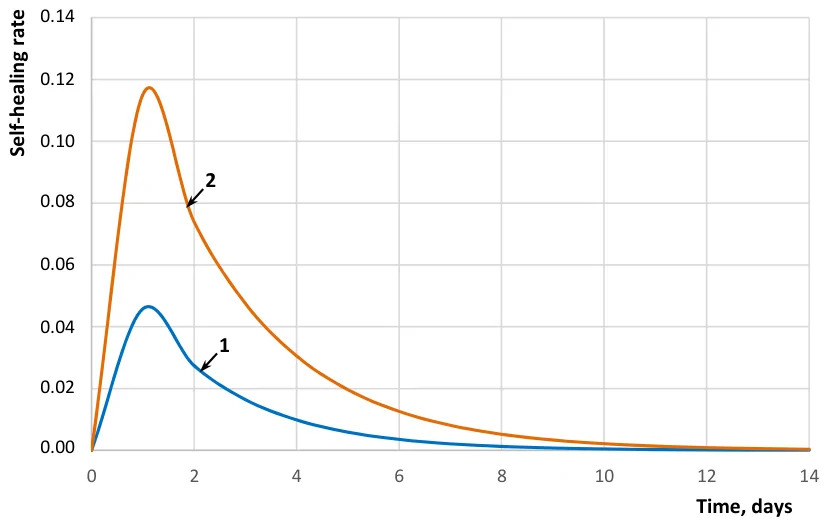
Change in the self-healing rate of asphalt concrete of ordinary composition (1) and with capsules (2).

For asphalt concrete containing capsules with AR-polymer, the increase in strength after self-healing is 38.8% of the residual strength (*SHI*(*t*) dependence in Figure 13), which ensures the healing of strength to 99.9% of its initial value (*HI*(*t*) dependence in Figure 13). For asphalt concrete of the control composition, the increase in strength is only 13.8% of the residual strength, which corresponds to 78.6% of its initial value. Assessment of the self-healing rate, which can be carried out using the formula:

$$\frac{dSHI\left(t\right)}{dt}=ab/e^{bt}$$

shows that the first day the rate of self-healing increases to maximum values, then asymptotically decreases to minimum values (Figure 14).

The intrinsic self-healing due to the properties of the bitumen matrix of asphalt concrete (control composition) realizes strength healing with a maximum intensity of 4.6% on the first day, and the use of capsules with an AR-polymer increases the self-healing rate by 2.5 times (up to 11.5%). A higher intensity of self- healing of asphalt concrete with capsules lasts up to 10 days.

The results of pilot testing prove the significant contribution of capsules with AR polymer to the ability of the material to resist mechanical stress due to self-healing. Further monitoring of the experimental sections will make it possible to assess the progress of these processes in real conditions of road surface operation.

The obtained results allow designing various types of asphalt concrete with capsules to extend the service life of pavements without repair. The obtained data are consistent with previously obtained results of studies on the fatigue life and self-healing of asphalt concrete with capsules [20,43]. The use of a polymer reserve inside the capsules allows for increased resistance to mechanical impacts, increasing durability.

## 4. Conclusions

Pilot testing of the technology for modifying the asphalt concrete mixture with capsules confirmed the possibility of creating self-healing asphalt concrete without changing the production chain. Compliance with the recipe requirements and temperature regimes for preparing the asphalt concrete mixture allows you to obtain self-healing asphalt concrete with capsules. The obtained results allow designing various types of asphalt concrete with capsules to extend the service life of pavements without repair.

Formation in the structure of asphalt concrete of A16U_L_ voids with a diameter 20–30% larger than the diameter of capsules allows to ensure 96% of their safety. Homogenization in production is sufficient to ensure homogeneity and uniformity of capsule distribution in a volume of 20 s. Only less than 5% of the capsules are located at a distance of less than 0.5 cm from each other, which can be considered not distributed.

Modification of asphalt concrete with A16U_L_ capsules with an AR polymer increases the self-healing of the compressive strength to 38.8% of the residual strength, which corresponds to 99.9% of the initial strength. The use of capsules with an AR polymer increases the rate of self-healing of asphalt concrete by A16U_L_ 2.5 times compared to the self- healing of asphalt concrete of the control composition.

Further development of the self-healing technology for asphalt concrete with capsules involves studying the long-term impact of pavement operating conditions on the capsules’ performance. Monitoring the experimental site will allow us to determine the degree of agreement between laboratory and field self-healing results, which will improve the design process for such mixtures.

## Figures and Tables

**Table 1:** Main properties of bitumen.

Parameter	Unit	Value
Penetration at 25 °C	0.1 mm	78
Ductility at 0 °C	cm	3.8
Ring and ball softening point	°C	48
Fraass breaking point	°C	−18
Mass change after aging	%	0.6
Change in softening point after aging	°C	7

**Table 2:** Composition of asphalt concrete A16U_L_.

Component	Content, %
Crushed stone	32.2
Crushed sand	33.8
Enriched sand	30.0
Mineral filler	4.0
Bitumen BND 70/100	4.19
Activator	6.6
Capsules with AR-polymer	5.6

**Table 3:** Main properties of asphalt with capsules.

No	Parameter	Standard Requirement	Value
1	Maximum Specific Gravity, g/cm^3^	–	2.574
2	Bulk Specific Gravity, g/cm^3^	–	2.493
3	Air Voids, %	2.0–4.5	3.1
4	Voids in Mineral Aggregate, %	not less 12	13.3
5	Voids Filled with Asphalt, %	72–85	72.2
6	Mean Rut Depth, mm	not more 6.5	5.89
7	Water Resistance Coefficient	not less 0.85	0.87

Notes. – there is no requirement of the standard.

**Table 4:** Physical properties of standard asphalt concrete mixtures.

Type of Mixture	State Standard	Content of Carcass Grains, % by Volume	Number of Contacts per Grain	Air Voids, %	VFA *, %
SMA-20	31,015	75–83	3.0	1.5–4.5	73.0–85.0
SMA-15		72–82	3.3	1.5–4.5	73.0
SMA-10		71–79	4.0	1.5–4.5	73.0–89.3
SMA22	58,406.1	65–80	2.9	2.0–6.0	63.3–63.5
SMA16		60–77	3.3	2.0–6.0	63.2–63.5
SMA11		60–75	3.8	2.0–5.5	65.4–65.8
SMA8		55–75	4.3	1.5–5.0	69.8–70.3
SMA-22	58,401.2	72–82	3.3	3.7–4.3	63.5–63.8
SMA-16		73–81	3.8	3.7–4.3	63.7–63.8
SMA-11		65–80	4.6	3.7–4.3	63.3–63.8
SMA-8		71–81	2.8	3.7–4.3	63.7–63.8
Type A	9128	62–72	4.7	2.5–5.0	65.2–65.3
Type B		52–62	5.3	2.5–5.0	64.9–65.0
Type C		40–52	6.1	2.5–5.0	64.5
A22U_L_	58,406.2	42–63	4.6	2.5–5.0	61.5–61.6
A16U_L_		42–63	5.3	2.5–4.5	64.0–64.2
A11U_L_		42–63	5.8	2.5–4.5	64.0–64.2
SP-32	58,401.1	10–60	4.5	3.7–4.3	57.6–58.5
SP-22		10–55	5.4	3.7–4.3	57.6–58.5
SP-16		10–52	6.5	3.7–4.3	57.6–58.5
SP-11		10–42	8.0	3.7–4.3	57.6–58.5
SP-8		10–34	11.1	3.7–4.3	57.6–58.5
SP-4		0–10	20.4	3.7–4.3	57.6–58.5

Note. * is calculated for bulk specific gravity *G_mb_* = 2.4 g/cm^3^.

**Table 5:** Results of calculation of parameters of the structure of the mineral framework of asphalt concrete.

Type of Mixture	State Standard	Parameters Value					
		*k_poly_*	*k_form_*	*k_bit_*	*k_pack_*	*k*	*d*_voids_, mm
SMA-20	31,015	0.62–0.75	0.66	0.65	0.84	0.22–0.27	1.24–1.53
SMA-15		0.61–0.68	0.66	0.65	0.84	0.22–0.24	1.16–1.20
SMA-10		0.68–0.78	0.66	0.65	0.84	0.24–0.28	0.52–0.66
SMA22	58,406.1	0.58–0.66	0.66	0.72	0.84	0.23–0.26	2.07–2.08
SMA16		0.56–0.63	0.66	0.72	0.84	0.22–0.25	1.51–1.61
SMA11		0.58–0.63	0.66	0.70	0.84	0.23–0.25	1.43–1.54
SMA8		0.60–0.70	0.66	0.67	0.84	0.22–0.26	0.93–1.00
SMA-22	58,401.2	0.52–0.68	0.66	0.71	0.84	0.21–0.27	1.87–2.01
SMA-16		0.59–0.69	0.66	0.71	0.84	0.23–0.27	1.21–1.37
SMA-11		0.62–0.67	0.66	0.71	0.84	0.25–0.27	0.86–1.01
SMA-8		0.57–0.77	0.66	0.71	0.84	0.23–0.30	0.78–0.96
Type A	9128	0.51–0.61	0.66	0.70	0.84	0.20–0.24	0.81–1.88
Type B		0.43–0.52	0.66	0.71	0.84	0.17–0.20	0.87–1.89
Type C		0.38–0.41	0.66	0.71	0.84	0.15–0.16	0.88–1.92
A22U_L_	58,406.2	0.37–0.46	0.66	0.73	0.84	0.15–0.19	2.00–2.64
A16U_L_		0.36–0.48	0.66	0.71	0.84	0.16–0.19	1.39–1.89
A11U_L_		0.45–0.51	0.66	0.71	0.84	0.18–0.20	1.24–0.98
SP-32	58,401.1	0.37–0.46	0.66	0.75	0.84	0.17–0.28	2.47–3.11
SP-22		0.39–0.48	0.66	0.75	0.84	0.17–0.27	1.80–2.36
SP-16		0.45–0.51	0.66	0.75	0.84	0.17–0.26	1.30–1.74
SP-11		0.41–0.67	0.66	0.75	0.84	0.16–0.23	0.96–1.31
SP-8		0.40–0.64	0.66	0.75	0.84	0.15–0.20	0.60–0.96
SP-4		0.40–0.62	0.66	0.75	0.84	0.13–0.23	0.32–0.79

Note. Ranges are used for compositions of grain materials equal to the permissible lower and upper boundaries in accordance with the standard.

**Table 6:** Particle size distribution of the control asphalt concrete mixture.

Grain Composition (Finer Than a Sieve Mesh, mm), %												
20	15(16)	10(11.2)	8	5	4	2.5	2.0	1.0	0.63	0.316	0.16	0.071
100	99	90	75	57	52	44	40	30	23	14	9	5

## Data Availability

The original contributions presented in this study are included in the article. Further inquiries can be directed to the corresponding author.
